# Online Signal-to-Noise Management for Evoked Potentials—Assessing and Explaining Response Quality

**DOI:** 10.3390/bioengineering13091008

**Published:** 2026-08-29

**Authors:** Gerald Fischer, Maria E. Holzknecht, Jens Haueisen, Daniel Baumgarten, Markus Kofler

**Affiliations:** 1 Institute of Electrical and Biomedical Engineering, UMIT TIROL—Private University for Health Sciences and Health Technology, Eduard Wallnoefer Zentrum 1, 6060 Hall in Tirol, Austria; 2 Department of Neurology, Hochzirl Hospital, 6170 Zirl, Austria; 3 Institute of Biomedical Engineering and Informatics, Technische Universität Ilmenau, Gustav-Kirchhoff Str., 98693 Ilmenau, Germany; 4 Department of Mechatronics, University of Innsbruck, Technikerstraße, 6020 Innsbruck, Austria; daniel.baumgarten@uibk.ac.at; 5 Department of Neurology, Medical University Innsbruck, 6020 Innsbruck, Austria

**Keywords:** electroencephalography, high-frequency oscillations, real-time signal processing, signal-to-noise ratio, somatosensory evoked potentials, waveform averaging

## Abstract

(1) Background: Evoked potentials (EPs) are an elegant, non-invasive, and reliable technique for assessing the functional integrity of neural pathways. They are, however, often limited by the difficulty and time needed to consistently distinguish signal from noise. (2) Methods: We have recently proposed a novel technique based on spectral domain evoked-to-background ratio (EBR) that enables fast data acquisition with online feedback about actual signal quality utilizing state of the art analog-to-digital conversion. Furthermore, we have developed a novel model-based signal-to-noise management concept allowing for suppression of biological and technical interference (swallowing, stimulation artifacts, electropolarization, and powerline potentials) and for online assessment of signal-to-noise ratio (SNR) for EPs. In this work, we experimentally confirmed this concept in ten healthy volunteers by investigating cortical EPs and high-frequency oscillations (HFOs) following median nerve stimulation. (3) Results: Both mathematical model and human data demonstrate that spectral target-band EBR governs the progress in SNR with increasing sweep count. For cortical EPs, SNR exceeded 10 dB beyond 90 averages in all participants. An SNR > 20 dB documented excellent signal quality and reproducibility. For HFOs, the SNR shifted to lower values by 12 dB, displaying pronounced individual variation, however, with smaller variation of HFO-band background activity (1.9 vs. 7.6 dB between the 25% and 75% percentile). Thus, individual HFO responses are more important for actual signal extraction compared to background activity. In subjects displaying a high HFO amplitude, reproducibility was confirmed for less than 1000 sweeps. (4) Conclusions: The present investigations confirm that individual EBR is the major factor defining SNR. Background noise can be reduced to a negligible level. Online assessment of background activity will allow for the most accurate moment-to-moment visualization of raw signal quality. This will facilitate termination of the data acquisition and may be based on quantified signal quality rather than predefined sweep count.

## 1. Introduction

Till this day it has remained challenging to distinguish low-amplitude somatosensory evoked potentials (SEPs) from physiological background activity that is not stimulus-locked. *N*-Interval Fourier Transform Analysis (*N*-FTA) allows for studying the power spectral density (PSD) of evoked and background activity separately but simultaneously by analyzing signal segments of a few minutes duration [1,2]. This double spectral investigation technique takes advantage of state-of-the-art biopotential amplifier technology. ΣΔ-analog-to-digital converters (ADCs) allow for full digital processing of neuropotentials [3,4] by digitizing the full bandwidth from 0 Hz to the Nyquist frequency.

These benefits can be translated into a novel signal-to-noise management (SNM) concept for evoked potentials. By selection of spectral target bands, SNM allows for assessing both clinical routine SEPs and evoked high-frequency oscillations (HFOs). Our SNM maintains trial averaging as the backbone of target-signal extraction, providing on top a number of noise suppression features tailored to the particular time or frequency domain properties of artificial disturbances like power line interference or stimulation artifacts. *N*-FTA allows for the definition of target bands which provide a high evoked-to-background ratio (EBR, i.e., the ratio of the evoked divided by the background PSDs). Zero-phase filtering [5] allows for extracting the target-signal components free of time shifts, therefore maintaining latency as a diagnostic key parameter [6].

So far, guidelines recommend repetition of each series [7,8] for confirming reproducibility at the cost of lengthy procedures. In contrast, recent guidelines for intraoperative SEP monitoring recommend signal-to-noise ratio (SNR) estimation for verifying the data quality of an individual series [9] based on a modified even/odd averaging algorithm developed by McDonald et al. [10]. Our SNM concept adopts this SNR assessment for clinical routine SEP recordings and HFOs with a single modification. In recent studies we have shown that long latency segments $T_{N}$ provide an accurate estimate of the background noise level for HFOs following median nerve stimulation [2,11]. In these segments $T_{N}$-tailored target-band filtering suppresses the remaining evoked signal components. Thus, the signal components passing the filter provide an estimate of the background activity.

This study investigates the primary cortical response (N20-P25 deflections) and HFOs following median nerve stimulation. Evoked HFOs are fast ripples of small amplitude (<0.5 μV) superimposed on the N20-P25 segment [6,12,13,14]. Thus, this test setting provides a condition in which small, evoked target waves must be separated from a dominant evoked component. Notably, HFOs display a remarkable variability in amplitude even in normal subjects [2,13]. This may cause a broad spectrum of variation in the test data. The literature provides good consistency for the definition of the target band (lower corner frequency 400 to 450 Hz; upper corner frequency 750 to 800 Hz [2,13,15]).

We hypothesized that basing SNM on full digital signal processing allows for effective online suppression of artificial interferences and online assessment of signal quality parameters. Thus, the major goals of this study were as follows: the development of (1) tailored processing blocks for optimal suppression of each single artificial noise component and (2) of an online-capable design maintaining latency and providing SNR estimates compatible with current guidelines [9]. Furthermore, we provide an identification and validation of key factors defining the progress of the SNR with incremental sweep count [16,17].

## 2. Materials and Methods

### 2.1. Participant Details and Experimental Protocol

In total, ten adult volunteers (four females; six males) without any known neurological disease were included (see Table 1). Here, data were merged from two studies which had slightly different scopes. Both studies aimed to collect data during right median nerve stimulation for studying *N*-FTA [1,2]. In the first study (subjects S1 to S4) only a very basic online visualization of unfiltered evoked potentials was available. Here, recordings were always terminated after 4 min. While *N*-FTA investigations of volunteers S1 to S3 were previously published [1,2], the current study focuses on SEP analysis of the data. In the second study (subjects S5 to S10) an online visualization of signal traces (both short-latency SEPs and HFOs) and the SNR were available. Again, minimum recording time was 4 min, but each series was allowed to be extended up to 6 min to further improve HFO quality. Thus, sweep count in an individual series varied between approximately 600 and 1500 averages. The experimental protocols were approved by the local institutional *Research Committee for Scientific Ethical Questions*.

### 2.2. Data Acquisition Hardware and Stimulation Parameters

**Figure 1:**
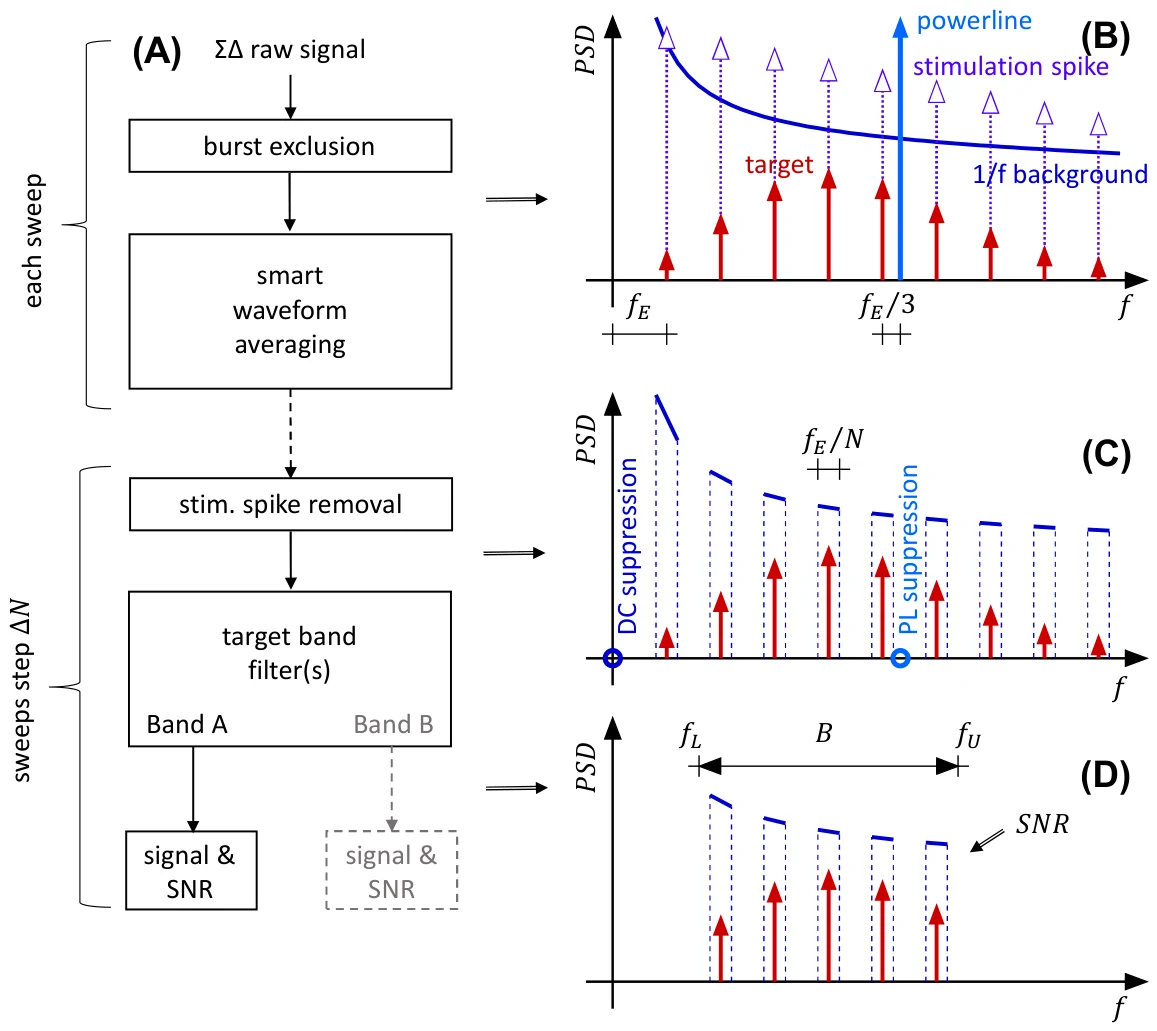
(**A**) Block diagram of the signal-to-noise management concept. At first, sweeps containing single isolated artifacts (e.g., swallowing) are removed. Smart waveform averaging allows for high direct current (DC) and powerline (PL) artifact suppression. The dashed central arrow indicates that processing is performed only for selectable sweep counts. The output of the smart averaging block allows for accurate removal of the stimulation spike and the exclusion of settling effects in the proceeding band-pass filter. Optionally, multiple target bands can be investigated at the output (gray block). (**B**) Spectral pattern after exclusion of muscle bursts (i.e., stationary and continuous background activity). Spectral components occurring at distinct frequencies (i.e., Dirac pulses in the frequency domain) are indicated by red arrows. (**C**) Spectral pattern before target-band filtering. (**D**) Spectral pattern after filtering of a single target band. The power of the evoked spectral pulses and the remaining background combs (both within the filter pass band) define the SNR of the processed evoked response. An SNR estimate can be displayed online together with the signal trace. See text for details.

The right median nerve was stimulated with a bar electrode with two 1 cm diameter stainless-steel contacts separated by 3 cm (Spes Medica S.r.l., Genova, Italy), strapped to the wrist. Monophasic pulses (200 μs duration) were generated by a DS7A neuro-stimulator (Digitimer Ltd., Welwyn Garden City, UK). A digital function generator (AFG-2225, Good Will Instrument Co., Ltd., New Taipei City, Taiwan) was used for triggering stimulation at a selectable constant frequency. For effective attenuation of powerline interference, we chose two distinct stimulation rates $f_{E}$, 2.46 Hz and 3.95 Hz, both in a common clinically applied range [7]. These two rates were originally introduced in [1]. Stimulation near 4.0 Hz is frequently applied for HFO recordings since they require a large sweep count [6]. At lower rates near 2.5 Hz the short latency response (N20 and P25 peaks) displays more pronounced amplitudes. The chosen rates provide a distinct $f_{E}\;/\;3$ displacement of both the 50 Hz interference and its 100 Hz overtone from the harmonics of the evoked response (see Figure 1B) and, thus, good suppression of both disturbances. Gold-plated Genuine Grass cup-electrodes (Natus Neurology, Galway, Ireland) were used to record SEPs from the C3’-Fz derivation. Signals were digitized at 9600 Hz using a g.USBamp (g.tec GmbH, Graz, Austria) biopotential amplifier. Not all subjects underwent all tests (see Table 2).

### 2.3. Signal Processing Pipeline and Algorithms

#### 2.3.1. AD-Conversion

The SNM concept (see Figure 1) presented in this study took advantage of a 24-bit ΣΔ analog-to-digital converter (ΣΔ-ADC, see [3]) operating at a 9.6 kHz sampling rate. Internal oversampling and noise-shaping of ΣΔ-ADCs kept the stochastic amplifier noise to a low level [4].

#### 2.3.2. Burst Exclusion

Sweeps containing occasional high-amplitude muscular bursts, like, e.g., swallowing artifacts, were automatically excluded at the beginning of the signal processing pipeline. Above 400 Hz, such myo-artifacts can be distinguished well from the stationary background activity. Thus, the applied detection algorithm analyzed the high-frequency band as described in the Online Resource S1, Section S1.3.

Figure 1B depicts the spectral distribution of the target signal and the multiple superimposed noise sources. A qualitative spectral pattern is shown, which is based on our previous analysis [1]. The evoked response is contained within a target band and displays the harmonics of the stimulation rate. Irregular background activity shows a continuous distribution with an 1/f-characteristic. Also the stimulation artifact contains the harmonics of the evoked rate. Power line interference occurs at 50 Hz or 60 Hz, depending on geographic location.

#### 2.3.3. Smart Trial Averaging

To meet online requirements, waveform averaging was applied early in the processing pipeline. Here, the required operations were executed in each sweep with relatively little computational demand. The implementation of the smart trial averaging block is described in detail in the Online Resource S1, Section S1. Briefly, the key features are the following: (a) automatic baseline correction, (b) high suppression of power line interference, and (c) extension of the time frame by two intervals which allows for filter settling outside of the evoked window. For high power line suppression, a stimulation rate was chosen such that the distance between the grid frequency and the nearest evoked harmonic equals $f_{E}\;/\;3$. Thus, 2.46 Hz and 3.95 Hz were selected as stimulation rates in this study.

In the frequency domain, waveform averaging acts as a comb filter [11,16], allowing frequencies near the evoked harmonics [18,19] to pass while suppressing frequencies between the harmonics. In Figure 1C this is schematically indicated. Furthermore, smart averaging effectively suppresses power line interference and near-DC electrode potentials.

The subsequent signal processing was optimized for speedy computing and fast data acquisition and visualization. To this end all remaining processing steps were not performed for each single sweep but after blocks of ΔN=15 sweeps. This drastically reduced computational load and is indicated by a dashed arrow in Figure 1A.

#### 2.3.4. Stimulation Spike Cancellation

The auto-covariance method [17,20] was applied to obtain an infinite impulse response (IIR) model of the transient decay of the artifact. This IIR model was subtracted from the data similar to that described in [21] (Online Resource S1, Section S2). The subtraction of the stimulation spike from the waveform in the time domain removes the interference at the harmonics in the frequency domain (Figure 1C).

#### 2.3.5. Band-Pass Filtering

In the final step a target band with a high EBR is selected (Figure 1D). As it is shown in Section 2.4 the mean EBR in the band relates to the SNR. Depending on the application multiple target bands can be investigated independently. For extracting the short latency deflections (N20-P25 segment) and the HFOs, two different zero-phase IIR filters were used (see Online Resource S1, Section S3). The short-latency SEP filter was a 20 to 264 Hz band pass of 2× 4th order. The selected HFO filter was described previously in [6]. The double application of the filter kernel as required by zero-phase IIR filters yielded a 419 to 765 Hz pass band with a sharp transition from the pass band to the stop bands. The concept of applying target-band filtering as the last noise suppression step allowed for efficient simultaneous computation of multiple target bands.

### 2.4. Expected SNR

The mathematical theory predicting the expected SNR from the signal’s spectral properties is described in detail in [11]. Briefly, the target-band SNR of a stationary series (i.e., a recording containing *N* sweeps of an evoked signal at a constant noise floor) is determined by two parameters: firstly, the ratio of the response window $T_{W}$ and the evoked cycle length $T_{E}$ (i.e., the time window ratio TWR), and secondly, the mean evoked-to-background ratio $\bar{EBR}$. Here, $\bar{EBR}$ is defined in the spectral domain (*N*-FTA). Conversion of both ratios to dB is denoted by $TWR_{dB}$ and ${\bar{EBR}}_{dB}$.

**Figure 2:**
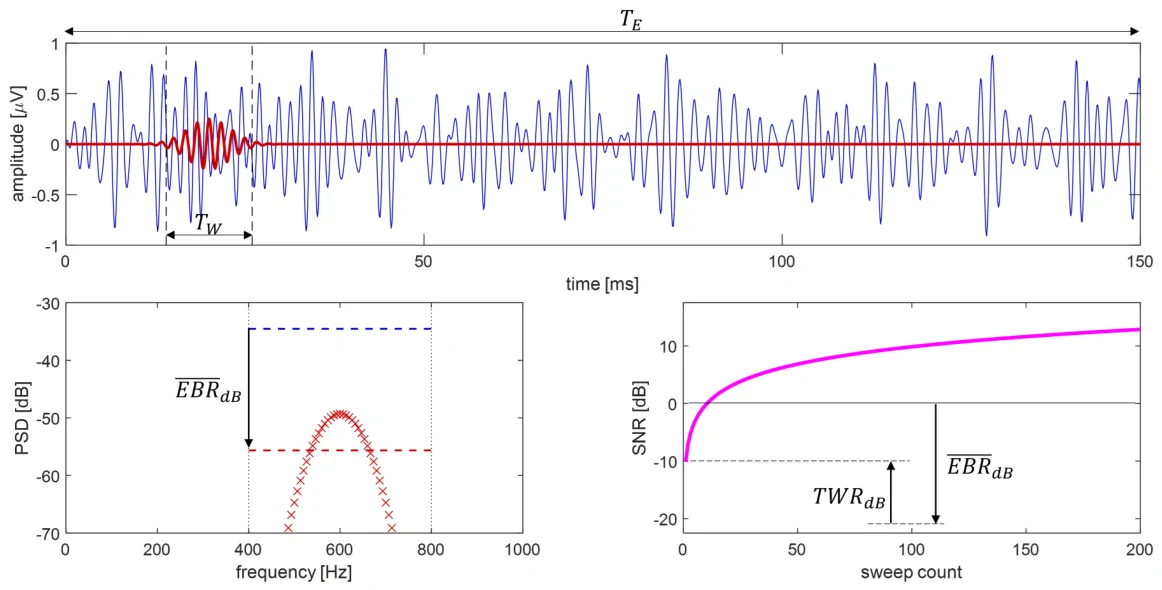
Schematic illustration of factors influencing HFO target-band SNR (400 to 800 Hz bandwidth). (**Top**): Within an evoked cycle ($T_{E}$) an evoked HFO response (red) is superimposed by band-pass filtered stochastic noise (blue). The response is contained in a time window $T_{W}$. (**Bottom Left**): Target-band filtered background noise is approximated by a constant PSD level $\bar{b}$ (blue dashed line) occurring only within the filter’s pass band. From the PSD of the evoked HFO (red markers) its mean background PSD $\bar{e}$ (red dashed line) is obtained (mind the logarithmic dB scale). The mean EBR is indicated by an arrow. (**Bottom Right**): With increasing sweep count *N*, trial averaging increases target-band SNR (in dB) in a logarithmic fashion. The initial offset (at N=1) is determined by the mean EBR and the ratio of time windows $T_{E}$ and $T_{W}$.

Figure 2 depicts the target-band SNR model. While mean power of the background activity $\bar{B}$ is approximately evenly distributed over the entire stimulation interval $T_{E}$, the mean power of the evoked HFO-response $\bar{E}$ is contained in the more narrow time window $T_{W}$. As demonstrated in [11], the expected value for the SNR obtained at *N* sweeps can be estimated from

(1) $$SNR_{dB}\left(N\right)≃TWR_{dB}+{\bar{EBR}}_{dB}+10\log_{10}N.$$

The expected value is a statistical prediction. The parameters $TWR_{dB}$ and ${\bar{EBR}}_{dB}$ define an initial offset of the SNR trace. Since evoked PSD is usually smaller than the background, ${\bar{EBR}}_{dB}$ is typically a negative value. Thus, it shifts the SNR trace to lower values (see Figure 2). The shape of the SNR trace is defined by the sweep count *N*. Importantly, Equation (1) is based on the assumption that background activity is a stationary process. Isolated events, like, e.g., occasional swallowing artifacts, were automatically excluded from the analysis by SNM.

### 2.5. Observed SNR

Online estimation of target-band SNR requires reliable estimates of actually observed power levels in averaged responses. An approach for assessing power levels is described in [11]. Briefly, two time windows are investigated. The power of evoked HFOs is distributed unevenly within a cycle $T_{E}$ and almost entirely contained in the narrow evoked response window $T_{W}$. However, this window $T_{W}$ contains also a superposition with background activity. By taking advantage of stationary background conditions, a second time window $T_{N}$ (noise window) is applied to obtain an independent estimate of the background power. Analysis of both time windows allows for computing the actually observed SNR [11]. The implemented SNM software processes only positive SNR values (in dB). In other words, SNR is only displayed if the evoked power exceeds the noise level.

Two strategies are applied for selecting the target-window $T_{W}$ for SNR estimation.

#### 2.5.1. Online Application

For simulating online applications, an automatic selection of the target window was implemented. Here, the latency of the N20 peak is determined at each processing step as an initial estimate for the location of the HFOs. In a next step, the envelope of the HFO trace was computed as described in [2] and analyzed in a ±3 ms window centered at N20. This maximum defines the center of the target window. Then, a width of ±3 ms is used for choosing $T_{W}$. For basing this analysis on a sufficiently accurate short-latency response, the actual SNM concept used automatic window selection for a sweep count with N≥90 averages.

#### 2.5.2. Comparison of Expected and Observed SNR

The computation of the experimentally observed SNR depends on the choice of the target window. For basing this analysis on most accurate assumptions, the target windows of individual subjects were manually selected at the end of each series (i.e., at a high SNR). Then, computations were repeated using the manual selections.

## 3. Results

**Figure 3:**
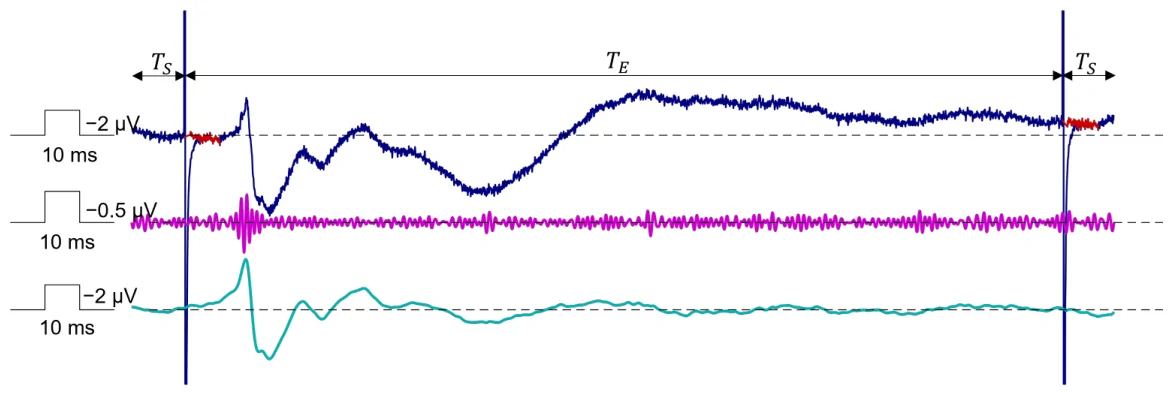
Example signal traces obtained at the block outputs in Figure 1 (Subject S4; 450 sweeps). Smart averaging (dark blue trace) extracts a noisy signal containing two stimulation artifacts (example output at dashed line in Figure 1). Stimulation spikes are removed by the next block (red segments). In the filter block the applied target band determines the extracted SEP. The N20-P25 deflections are obtained by transferring frequencies in the β to high-γ-bands [1] (cyan trace). HFOs are obtained by band passing the high-frequency band (magenta trace) [2]. Importantly, the filter is allowed to settle within the two shutter intervals $T_{S}$ keeping the evoked cycle $T_{E}$ free from settling effects. See also the text.

All ten subjects tolerated the recording sessions without problems. For investigating data segments containing at least N=1000 sweeps, we performed the following analysis: for the subjects S1 to S8, the series obtained at a stimulation rate $f_{E}$ = 3.95 Hz were analyzed. For the subjects S5 to S10, the data from two series at $f_{E}$ = 2.46 Hz were merged to yield more than 1000 sweeps per investigation. Thus, in total 14 investigations were available. In four subjects, S5 to S8, the comparison of HFOs obtained at two different stimulation rates was possible. Figure 3 depicts example signal traces obtained during the processing steps of the SNM pipeline.

In the 14 series combined, N20 latency was 19.7 ± 1.2 ms (mean ± standard deviation) and displayed a Pearson correlation of 0.72 with body height. Median N20-P25 peak-to-peak amplitude was 5.0 μV (2.1 to 10.4 μV). In all cases, short-latency SNR exceeded 10 dB at the highest sweep count indicating good reproducibility.

The median peak-to-peak HFO amplitude was 0.28 μV with a maximum of 0.71 μV. In three series the HFO SNR did not exceed 5 dB reflecting low response amplitude. Thus, in these series evoked HFOs could not be reliably distinguished from background noise making the assessment of a minimal amplitude unreliable. For spectral analysis, two series containing multiple swallowing artifacts were excluded after visual inspection. The median background level in the remaining twelve series was −24.3 dB (−24.9 to −23.1 dB).

In the absence of online SNM in the earlier study, two subjects (S3 and S4) out of four displayed a N20-P25 peak-to-peak amplitude below 3.0 μV. Lack of sufficient online visualization in combination with a low sensory perception threshold (and thus a low stimulation intensity) may have contributed to the relatively low amplitude responses. Online assessment of SEPs in the second study had a positive impact on data quality. Here, only one subject (S7) out of six displayed a N20-P25 peak-to-peak amplitude below 3.0 μV.

### 3.1. Artificial Noise

For quantifying the level of all kinds of artificial noise superimposed on the recordings, we analyzed them as described in the Online Resource S1, Section S4.1. Below, results are presented in the format median value [minimum maximum]. The burst exclusion criterion (see Online Resource, Section S1.3) reliably eliminated isolated artifacts. Such events were rare. More than 99% of all sweeps were used for further analysis.

DC-offsets caused by electrode polarization were 8.1 [−24.1 21.4] [mV]. The polarity of the polarization voltage was random (in four subjects out of ten a negative polarity was observed). The peak-to-peak powerline interference was 4.3 [2.3 14.5] [μV]. Highest power-line interference was observed in subjects S3 and S9 (14.5 and 14.0 μV, respectively). The peak-to-peak amplitude of the stimulation artifact was 860 [120 7400] [μV].

Amplifier noise was assessed in a baseline recording (shortcut inputs) and was −27 dB. This value was a few dB below the *N*-FTA HFO-band background levels listed above. Furthermore, between 600 Hz and 650 Hz, the ΣΔ-ADC in the amplifier also generated a single narrow idle-tone spectral peak in the noise floor. The peak frequency slightly varied between recordings but was observable for most recordings both for shortcut inputs and regular recordings. Since such peaks may reflect artifacts generated by ΣΔ-ADCs (idle-tones), they were excluded from analysis when computing mean background power levels.

### 3.2. SNR Estimates

**Figure 4:**
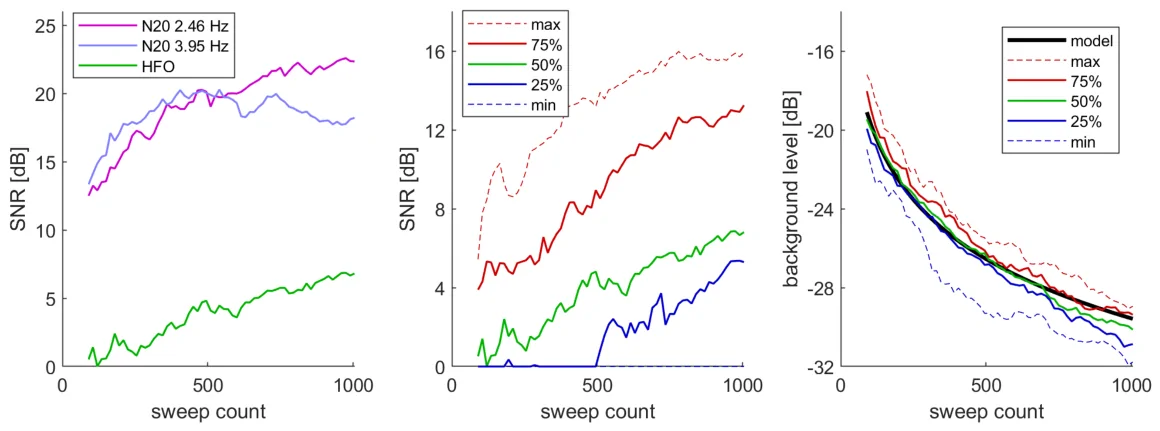
(**Left**) Log-shaped progress of median SNR over sweep count. For the N20-P25 the median SNRs obtained at the two studied stimulation frequencies are shown. For the HFO signals the median of all 14 series is shown. Below approximately 500 sweeps the N20-P25 SNR is approximately 12 dB above the HFO SNR. At higher sweep counts this offset is maintained only at the lower stimulation rate (plateau at high rate, see text). (**Middle**) SNR estimates as a function of sweep count display a relatively large distance between percentiles (25% steps) of several dB. (**Right**) Residual background noise level as a function of sweep count. Distance between percentiles is in the order of a few dB. The experimentally obtained traces are in good agreement with model predictions (see also text).

The left panel of Figure 4 depicts the median SNRs obtained for the N20-P25 segment and for the HFOs. For the short-latency deflections, a systematic influence of the stimulation rate was observed. Here, at the faster rate a plateau was observed at high sweep counts. At the lower rate, an increase in SNR was observed for the entire range. A comparable morphology of SNR progression was observed for HFOs but shifted downwards by more than 10 dB. Here, improvements at high sweep count were observed with both stimulation rates, and thus data were merged. In the middle panel of Figure 4 percentile levels of SNR for the 14 investigations are shown. The automatic determination of the target window was applied. Levels of high SNR (max and 75% percentiles) coarsely reflected a log trace with superimposed statistical variations. Lower SNR levels (25 and 50% percentiles) appeared shifted downwards by an offset, which was relatively large. At N=1005 the distance between the 25% and 75% percentile was 7.6 dB. Background levels (right panel of Figure 4) displayed a log-shape of opposite orientation, and percentile levels were spaced much closer. At N=1005 the distance between the 25% and 75% percentile was only 1.9 dB. The plot also contains a model prediction of background power over sweep count. Here, a background power $\bar{B}$ was set to the median value reported above (−24.3 dB).

**Figure 5:**
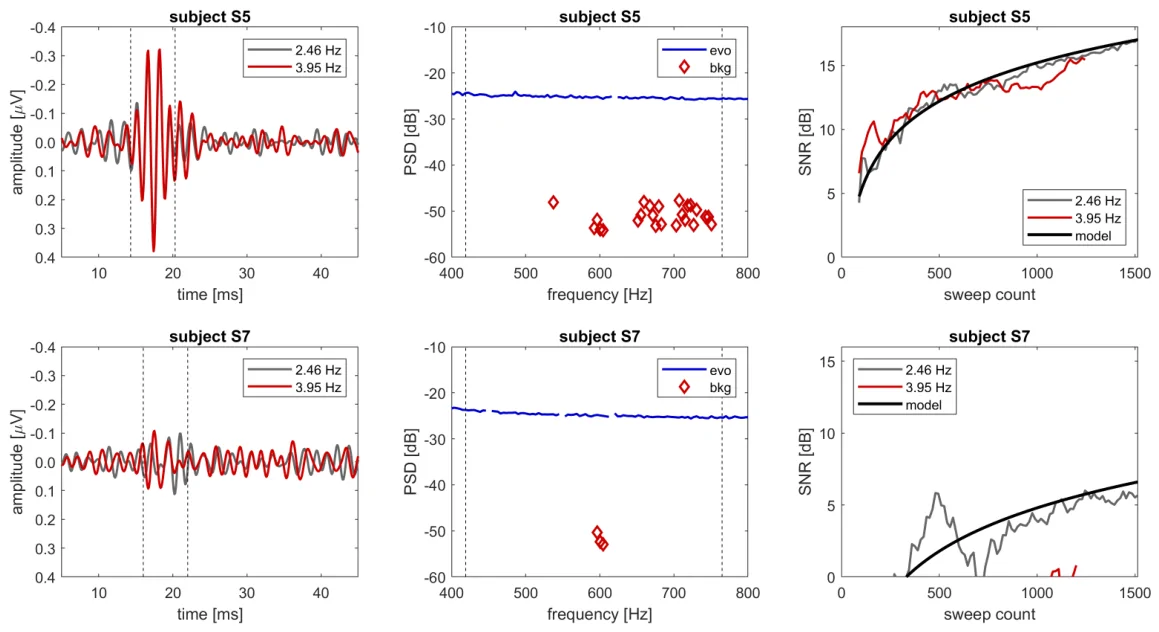
Comparison of data in two subjects ((**top row**): subject S5, (**bottom row**): subject S7). The left panels depict the HFOs at maximal sweep count (1200 to 1500) at two stimulation rates. The dashed lines mark the segment $T_{W}$ chosen for SNR estimation. The middle panel depicts PSD as obtained by *N*-FTA at the stimulation rate $f_{E}=$ 3.95 Hz. The blue lines depict the background PSD, while the red diamonds reflects the evoked PSD. Spurious background peaks caused either by harmonics of the line frequency or idle tones were removed from the background traces. The dashed lines mark the corner frequencies of the target-band filter. The right panels show log-shaped SNR progress with increasing sweep count *N* for the two stimulation rates. Furthermore, also the expected SNR as computed from (1) is shown. The mean background and evoked power were obtained from the *N*-FTA (see text).

Results for two individual subjects investigated at both stimulation rates are shown in Figure 5. For illustration purposes, one participant displaying a high SNR (subject S5) is contrasted with one individual with a low SNR (subject S7). For subject S5, the SNR was above 15 dB, and HFOs were obtained with high reproducibility. For subject S7, the SNR was 1 and 6 dB for the faster and slower stimulation rate, respectively, indicating insufficient reproducibility. The high SNR in the data of subject S5 also allowed for detection of several evoked PSD components between 537 and 750 Hz. This permitted accurate estimation of the mean evoked and background PSD and, thus, accurate prediction of SNR development during stimulation at both rates. For subject S7 only a few evoked PSDs were detected. This resulted in a 10 dB-lower predicted SNR. However, due to the high noise levels no accurate estimate of mean evoked PSD could be obtained. Thus, SNR prediction was of limited accuracy.

A linear regression analysis was applied for correlating SEP and HFO voltage levels as defined in the Online Resource S1, Section S4.2. HFO amplitude was positively correlated to N20-P25 amplitude. Based on the coefficient of determination $r^{2}$, 58% of the evoked HFO-amplitude variation was related to the N20-P25 amplitude variation.

### 3.3. SNR and Reproducibility

**Figure 6:**
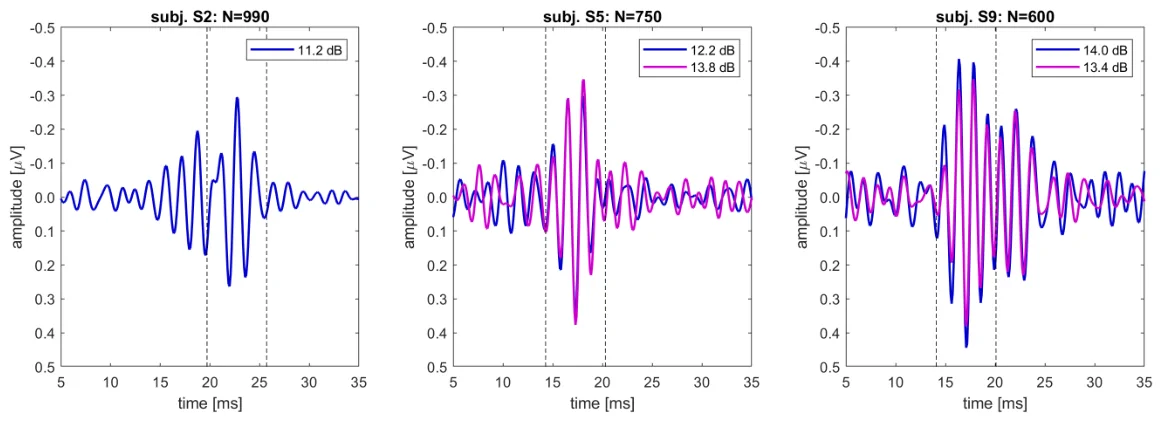
HFOs for selected recordings which exceeded an SNR of 10 dB within less than 1000 sweeps. The dashed lines indicate the automatically selected windows $T_{W}$. Subjects S5 and S9 allowed for comparison of two successive series at identical sweep counts. The coefficient of determination $r^{2}$ was computed within $T_{W}$ (see also text).

In subjects S5 to S10, two recordings with a 2.46 Hz stimulation rate were available. In subjects S5 and S9, the SNR exceeded 10 dB at the end of both series. Comparison of peak amplitudes and their latencies both for HFO signals and their envelopes revealed a deviation in all peak amplitudes well below 20% (Figure 6, Table 3). Deviation in latency was always within ±0.1 ms (i.e., the resolution of the sampling rate) except for the global minimum of the HFO trace in subject S9. Here, the deviation of 1.4 ms corresponds to one oscillation of the HFO cycle (see also Figure 6). In subject S9 two peaks in the envelope were found. In both subjects high coefficients of determination (>0.95) were obtained.

## 4. Discussion

We developed an online-capable SNM concept that effectively removes artificial noise or interference from the raw data. We demonstrated that physiological background activity is the dominating remaining source of interference. Only in the HFO band was the −27 dB amplifier noise level was close to the total background level (−24 dB at 600 Hz). Due to the 1/f spectral characteristic of cortical activity it clearly dominates up to the high-γ band. Quantification of the SNR in dB provides a robust measure for response quality being in widespread use in biosignal analysis [10,18].

The EBR determines the log-shaped progress of the SNR with incremental sweep count both for SEPs and evoked HFOs [11]. Our results are comparable with an earlier study on intraoperative SEPs [10] emphasizing the need to express the SNR as a power ratio of the evoked and background signals within a short-latency time window $T_{W}$. Furthermore, MacDonald et al. stressed the importance of converting the SNR to dB for a standardized interpretation of SNR values and proposed a level of 10 dB sufficient for attesting reproducibility in a single series [9,10]. At an SNR of 10 dB the power of the target signal equals ten times the power of the noise. Thus, at this level the evoked response is clearly the dominating component. Our study also confirms the feasibility of this SNR level for routine SEPs and HFOs.

While the current work focused on the evaluation of SEPs, the general underlying theory applies also to auditory and visual evoked potentials [19]. It may be of clinical use whenever time consuming repetitions of entire series should be avoided and proper filtering may serve for reducing the required number of sweeps per series. In this study, an initial prototype implementation of SNM was studied. Future routine application will require certified software.

### 4.1. Comparison of Target-Band SNRs

The proposed SNM was able to extract the N20-P25 component and HFOs simultaneously with high quality. For most of the N20-P25 potentials, the SNR well exceeded 10 dB within 100 sweeps. The N20-P25 response was extracted by selecting target frequencies extending from the β-band to the high-γ band. The selection of corner frequencies was based on previous spectral analysis [1] and was close to most recent recommendations [9]. Consistent with clinical experience, improvement in the SNR with increasing sweep count was clearly faster for the N20-P25 deflections than for the HFOs, and values were more than 10 dB larger. This advantage in assessing the primary cortical deflection can easily be explained: the target-signal amplitude of the N20-P25 response is more than an order of magnitude larger than the amplitude of HFOs. More precisely, the larger EBR in the β- and high-γ band [1] as compared to the HFO-band [2] determines this SNR shift quantitatively.

To achieve a reliable extraction of the N20-P25 component, we first collected 90 averages, before beginning the automatic analysis of HFOs. During the experiments, two participants reached the suggested reproducibility level of 10 dB [9] for the N20-P25 potential even within 15 s of recording. However, similar as for the HFOs, we also observed a pronounced variation in response quality for the N20-P25 component, emphasizing the need for an objective and quantitative online SNR assessment of all SEP traces.

At the faster stimulation rate, the SNR for the N20-P25 component reached a plateau around 500 sweeps and did not improve further, while it did so at the slower rate. Importantly, SNM assesses the noise level within the window $T_{N}$ at the end of each sweep cycle. Ideally, this window should not contain any evoked activity. At higher stimulation rates, however, the cycle shortened, which introduced a contribution of late responses to the power level estimate. These long-latency components were not fully suppressed by the target band filters and were, thus, unintendedly added to the noise estimates. Consequently, this effect caused an underestimation of the SNR and was more pronounced for high stimulation rates (i.e., at short evoked intervals) but was pronounced only at low noise levels (i.e., at high sweep counts). This drawback might be acceptable in most clinical routine settings, which may not require an SNR near or above 20 dB. For the SNR of HFOs, no such effect was observed, which might be due to the more than 20-times higher value of the lower corner frequency (leading to a more powerful attenuation of late responses). Lower stimulation rates (i.e., at longer evoked intervals), allow for a more accurate estimation of SNR levels: this led to an almost constant offset between the SNRs derived for N20-P25 vs. HFOs.

### 4.2. Signal-to-Noise Ratio Model

#### 4.2.1. Artificial Noise

Applying smart trial averaging early in the processing pipeline allowed for high initial attenuation of power line interference and electrode polarization. Power line attenuation was improved by selecting dedicated stimulation rates. The major part of electrode polarization was removed by baseline correction after averaging. Target-band filtering further suppressed these disturbances. The computation of the stimulus template *after* trial averaging provides several advantages. Smart averaging already attenuates all other kinds of noise. Thus, the estimation of the stimulus template is obtained from a cleaned data segment. In particular, accurate baseline correction is important when computing a pulse shape with an asymptotic decay. At each SEP update (steps ΔN) this allows for taking advantage of all previous sweeps. This yields increasingly accurate templates with increasing sweep count and, thus, an accurate cancellation of stimulation artifacts from the data. Thus, despite displaying relevant amplitudes in the raw data, the influence of all kinds of artificial noise on the SNR (and thus, also on the HFOs) was small.

#### 4.2.2. Spontaneous Activity

Background activity involves cortical, subcortical, and muscular potentials that are not time-locked to stimulation and are of irregular shape. Thus, they can be considered as mainly a stochastic activity. In the context of SEP recordings, they represent the dominant source of noise. Consequently, the SNR model requires only a single dominating source of noise: the mean background power $\bar{b}$ in the target band. A recently developed theoretical framework [11] predicts the log-shaped SNR improvement. This was confirmed by our experiments and agrees with previous experimental data [10]. For high amplitude evoked activity and stationary conditions, it was possible to accurately relate model predictions with the experimentally obtained SNR. At low evoked amplitude, power estimates made by *N*-FTA were less accurate (limit of evoked resolution [2]). However, model predictions and experimental data show that a large negative evoked-to-background ratio is the dominating factor causing low HFO-SNR. In conclusion, our data and methodological background suggest, that the difficulty to record HFOs in particular individuals may indeed be caused by the low amplitude response in those subjects and is not a matter of background noise.

Notably, the data presented by McDonald et al. on intraoperative SEPs provided comparable findings based on a vast amount of data and a 30 to 300 Hz target band [10] for cortical derivations. They also reported SNRs of other evoked responses, such as compound nerve action potentials and spinal cord potentials, suggesting that the same transfer of stochastic noise dominates most kinds of SEPs.

#### 4.2.3. Stochastic Noise Transfer

**Figure 7:**
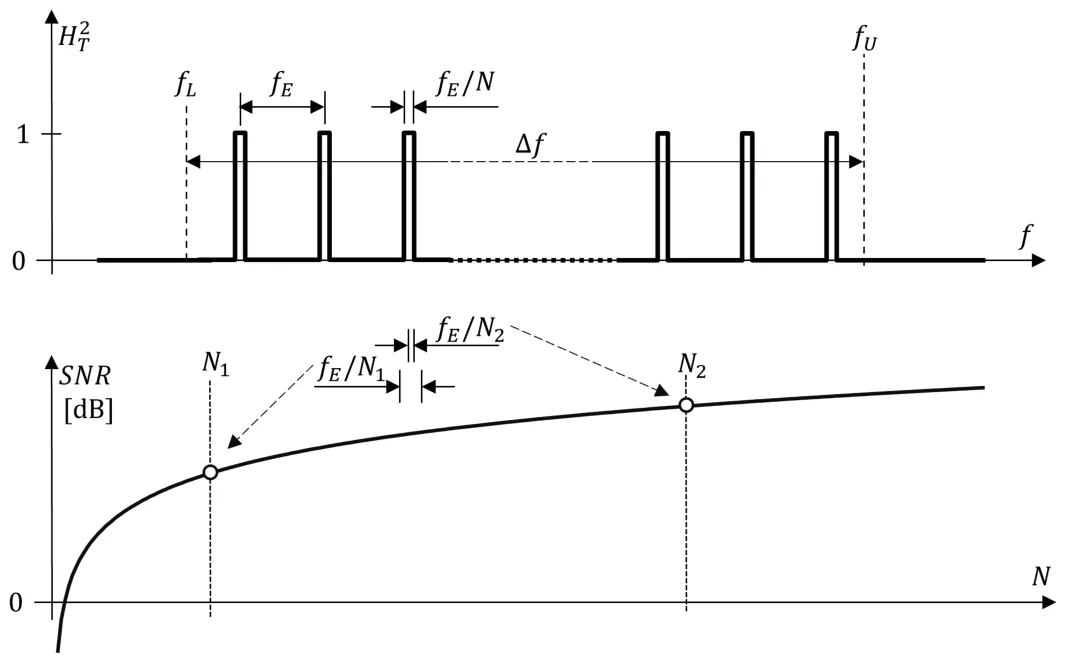
Schematic representation of stochastic noise transfer in the frequency domain. (**Top**): When applying trial averaging and target-band filtering in series (see Figure 1), a total transfer function $H_{T}$ is obtained. Its square $H_{T}^{2}$ allow for stochastic noise transfer. Outside the target band (below $f_{L}$ and above $f_{U}$), noise transfer is near zero. Within the target band the combs of the trial averaging transfer function define noise transfer. Their area is inversely related to sweep count *N*. (**Bottom**). When converting SNR to dB, the comb area is reflected by a logarithmic progress of response quality with increasing sweep count.

Figure 7 schematically illustrates the frequency domain transfer of the stochastic noise across the signal-processing pipeline. The combs of the trial averaging transfer functions are idealized as rectangles of width $f_{E}\;/\;N$. The trial averaging block is in series with the target-band filter of bandwidth Δf (see Figure 1). Below the lower corner frequency $f_{L}$ and above the upper corner frequency $f_{U}$, the target-band filter attenuates the combs. Notably, the finite-periodic evoked signal passes this transfer function without distortion. The broad-banded stochastic noise passes through the combs within the target band. This yields a total area Δf/N for noise transfer. Thus, the stochastic noise passes the signal processing pipeline within its pass band(s).

#### 4.2.4. Frequency Resolution

The finite width of the averaging combs can be interpreted as a finite frequency resolution obtained by a finite recording time. With increasing sweep count *N*, recording time increases, and uncertainty in frequency resolution decreases accordingly. When converting the SNR to dB, increasing frequency resolution is reflected by a log-curved progress of the SNR. Notably, the physiological signal levels may shift the log-curve up- or downwards, but its shape remains preserved. As a general principle in signal processing, small bandwidth (i.e., accurate frequency resolution) concurs with increasing time constants. This is known as the uncertainty principle of signal processing [22] which applies also to trial averaging. For high stochastic background levels being typical for evoked potential investigations, the limitation of frequency resolution by finite recording time is the major limiting factor for the suppression of background activity. Importantly, spectral analysis by fast Fourier transform (FFT) displays exactly the same frequency resolution. Thus, techniques using FFT in the context of response extraction [23,24] never managed to outperform or replace standard trial averaging.

### 4.3. Implications for Future SNR Improvements

The relation of frequency resolution and recording time is a fundamental physical principle which imposes restrictions on waveform averaging. Theoretically, the use of narrow target bands may allow for some improvement; however, there are evident limitations that can be anticipated from Figure 2. When reducing bandwidth, the mean evoked PSD $\bar{e}$ cannot exceed the highest evoked PSD (i.e., smallest negative value). A narrow bandwidth limits temporal resolution. Thus, the minimum bandwidth requirement combined with the smallest negative EBR value define the limitations for signal extraction for classical trial averaging. Future improvements might aim for maintaining phase consistency of brain responses [14]. For example, bootstrapping in the phase spectrum was proposed for reducing the number of sweeps required for obtaining a desired signal quality [25]. However, the adoption of the proposed online SNM concept to bootstrapping was beyond the scope of this work.

#### 4.3.1. Electrode Montages for High SNR

Given the restrictions mentioned above, MacDonald et al. [10] reported a remarkable finding for obtaining a practical improvement in the SNR. When extracting SEPs over the cervical spine, their standard reference electrode was the contra-lateral Erb’s point. This montage captures significant cardiac background activity. By placing the reference electrode at the mastoid, the background level is decreased by more than 10 dB, which in turn shifted the SNR trace by >10 dB upwards, thereby drastically reducing recording time. Optimization of reference placement might be of particular value for extracting responses of peripheral nerves and spinal cord potentials. When targeting somatosensory HFOs, EBR might be increased by placing the bipolar electrodes such that they are located over the maximum and minimum of the bipolar source pattern. This might theoretically deliver the highest SNR by using the maximal response amplitude.

#### 4.3.2. Response Amplitude and SNR

For a given signal morphology, power is proportional to the square of amplitude. Thus, the time needed for obtaining a certain SNR level (e.g., 10 dB) is inversely proportional to the square of the response amplitude. Thus, if the amplitude is halved, a four-fold recording time is needed at the same background level. This concurs with clinical experience. Recording of low-amplitude SEPs becomes easily unpractical.

#### 4.3.3. Background Level and Recording Quality

The background level displayed only small individual variations. In particular, in situations of low response amplitude, automatic monitoring of background levels might improve the usability of SEP recording. Several options for assessing background activity online are available: the use of a long latency interval $T_{N}$ as in this study, modified even/odd averaging as described in [10] or the evaluation of background data in the absence of stimulation (e.g., for control purposes prior to or after evoked potential acquisition). In the case of a missing or unclear response, the knowledge of the background level allows for computation of the resolvable evoked amplitude for a given sweep count. Thus, a sufficiently large response is detectable above this value. This may allow for justified termination of recording when a predefined minimal response amplitude is not achievable with a selected number of sweeps.

### 4.4. Online Capability

Online capability involves two aspects. Firstly, the ability to handle the data on the processor(s) with a sufficiently small temporal delay. Here, the computational performance of current computers allows for straightforward realization of this requirement. The algorithms described in this study can easily run online on a standard laptop. The second aspect is the organization of the program structure. For example, zero-phase IIR filters require analysis in the backward time direction. The program structure must be organized such that this applies only to relatively short data segments.

#### 4.4.1. Frame Based Processing

Linear time-independent filters and trial averaging both apply only linear operations. Thus, the commutative property can be used. Implementing trial averaging as the first block provides the advantage of filtering only relatively short frames at each evaluation. The temporal delay required for such a filter operation becomes negligible in an application that requires some minutes of investigation. Our implementation extends the evoked period by two adjacent time windows. Thus, the signal within the evoked cycle remains free of settling artifacts.

#### 4.4.2. Zero-Phase Filters

Digital filters allow for avoiding time shifts by implementing zero-phase filters [5]. Their use is mandatory when response latency is a diagnostic key parameter. This remarkable advantage of these digital filters appears to not be fully recognized in the field of SEPs. For example, current recommendations for intraoperative SEPs [10] apply IIR filters which induce time shifts. As can be taken from Figure 3 in those guidelines, a decrease in the upper corner frequency from 3000 Hz to 300 Hz introduces a prolongation in latency by more than 0.5 ms. In contrast, the here-proposed SNM concept maintains latency by using ΣΔ-ADs and zero-phase target-band filters.

#### 4.4.3. HFO Target-Band Filter

In the present study, we chose the zero-phase IIR HFO target band pass, which was originally proposed in [6], and we also had used it in [2]. Lanzone et al. [6] focused on the application of HFOs for monitoring the effect of anti-epileptic medication. We kept this promising clinical application in mind when we developed the presented technique. In the developmental phase of the SNM software (Version 2.10), we also tested other types of zero-phase filters. For example, double application of the Ricker wavelet [26] also provided a high pass of the fourth order and a good separation of the HFO component from the N20-P25 deflection [11].

## 5. Conclusions

Here, we present a novel SNM approach that takes advantage of state-of-the-art analog-to-digital conversion and thus allows for full digital signal processing. We developed and applied techniques which reduced all artificial noise components to a negligible level. The remaining dominant source of disturbance was the spontaneous physiological background activity (mainly from the brain). We developed a theoretical framework that identified the mean spectral EBR ratio [1,2] in the SEP target band as the dominating remaining interference. The EBR defines the recording time required for obtaining a desired signal quality. Our SNM approach allowed for assessing the SNR online. For stable experimental conditions these estimates accurately matched theoretical predictions. Furthermore, the approach permitted identifying varying conditions during investigations. This might support timely decisions on proper countermeasures and may significantly shorten clinical SEP studies in the future.

The presented SNR concept may serve to improve the future clinical usability of SEP recordings. Online assessment of the SNR may render the repetition of recordings for confirmation of reproducibility obsolete [10,11], as the continuous quantification of the SNR enables objective online estimation of signal quality. By assuming that avoiding repetitions of entire recording series saves half of the recording time and that improved signal processing (target-band filtering) saves another half of the remaining recording time, we estimate the time saving capabilities at about 75% compared to standard recommendations, not counting the time for mounting electrodes and changing stimulation sites. In summary, the technical developments presented here may thus help to reduce valuable time a patient needs to spend in the neurophysiology lab.

We applied this novel approach to both standard SEPs as well as HFOs following median nerve stimulation. The latter are challenging to extract due to their low amplitude, which displays pronounced inter- and intra-individual variations. The amplitude of individual responses was identified as the major factor accounting for difficulty in signal extraction.

In about one third of the participants, HFOs exceeded an SNR of 10 dB within less than 1000 sweeps. The results suggest that this quality level recommended by guidelines on intraoperative SEPs [9] might also apply to routine SEPs and HFOs. By taking advantage of this marker, repetition of recordings could be avoided in routine studies. Particularly in subjects displaying a low-amplitude response, this approach may serve as the method of choice for basing diagnostic interpretation on objective and quantitative quality markers, thus avoiding impractical time consuming repetitions.

## Figures and Tables

**Table 1:** Study population.

	Min	Median	Max
age [y]	27	29	60
height [cm]	165	177	190
weight [kg]	50	72	90

10 subjects (4 female).

**Table 2:** Applied stimulation series.

Stimulation Rate $f_{E}$	3.95 Hz	2.46 Hz
subjects S1 to S4	1	1
subjects S5 to S8	1	2
subjects S9 & S10	0	2

At least 4 min duration per series.

**Table 3:** Deviations at envelope peaks.

	Latitude			Amplitude		
	Series I	Series II	ΔLat.	Series I	Series II	Rel.dev.
	[ms]	[ms]	[ms]	[nV]	[nV]	[%]
**subject S5**						
global peak	17.3	17.4	−0.1	342	375	−9.1
maximum	17.3	17.3	0.0	345	377	−8.6
minimum	18.0	18.1	−0.1	−298	−347	−15.2
peak-to-peak	0.7	0.8	−0.1	643	724	−11.8
**subject S9**						
global peak	17.1	17.2	−0.1	443	379	15.7
local peak	22.2	22.1	0.1	262	256	2.4
maximum	17.1	17.1	0.0	444	381	15.3
minimum	16.4	17.8	−1.4	−408	−348	15.8
peak-to-peak	0.7	−0.7	−1.4	852	727	15.8

## Data Availability

The original data presented in this study is available for download at https://www.umit-tirol.at/page.cfm?vpath=departments/technik/iebe/forschung/signal-to-noise-management-snm (accessed on 30 July 2026).

## Supplementary Materials

The following supporting information can be downloaded at https://www.mdpi.com/article/10.3390/bioengineering13091008/s1.

## Glossary

ADC	analog-to-digital converter
EBR	evoked-to-background ratio
HFO	high-frequency oscillation
IIR	infinite impulse response
*N*-FTA	*N*-Interval Fourier Analysis
PSD	power spectral density
SEP	somatosensory evoked potential
SNM	signal-to-noise management
SNR	signal-to-noise ratio
$T_{W}$	time window evoked response
$T_{N}$	time window noise segment
