# Supplementary material for: Online Signal-to-Noise Management for Evoked Potentials—Assessing and Explaining Response Quality
Source: Bioengineering (Basel). 2026 Aug 29;13(9):1008. doi: 10.3390/bioengineering13091008 (PMC13603548; doi:10.3390/bioengineering13091008)
Supplement: Supplementary file 1 [file bioengineering-13-01008-s001.zip › bioengineering-4504722-Supplementary Materials.pdf]

Online Supplement for: Real-Time Signal-To-Noise Management for Evoked Potentials – Assessing and Explaining Response Quality

Gerald Fischer[1*], Maria E. Holzknecht[2], Jens Haueisen[3], Daniel Baumgarten[4], Markus Kofler[2, 5]

[1*] Institute of Electrical and Biomedical Engineering, UMIT Tirol – Private University for Health Sciences and Health Technology, Eduard Wallnöfer Zentrum, Hall in Tirol, 6060, Austria.
[2]Department of Neurology, Hochzirl Hospital, Hochzirl, Zirl, 6170, Austria.
[3]Institute of Biomedical Engineering and Informatics, Technische Universität Ilmenau, Gustav-Kirchhoff Str., Ilmenau, 98693, Germany.
[4]Department of Mechatronics, University of Innsbruck, Technikerstraße, Innsbruck, 6020, Austria.
[5]Department of Neurology, Medical University Innsbruck, Christoph-Probst-Platz, Innsbruck, 6020, Austria.

*Corresponding author(s). E-mail(s): gerald.fischer@umit-tirol.at;

This document provides the Online Supplement for the main manuscript [1] submitted to Bioengineering.

1 Smart Trial Averaging

Smart trial averaging adds additional noise suppression features to classical trial averaging which are described in this section.

1.1 Frame Selection

Smart trial averaging uses two shutter windows adjacent to the evoked interval. At each sweep a raw data frame is taken containing the evoked interval and the two shutters (see Figure 1). As for standard averaging, the frames are summed up and divided by the number of sweeps. This concept extends the length of the averaged response segment to a time interval which is longer than the evoked interval. The two adjacent shutters are used for baseline correction and provide a time segment for settling of the target-band filter. Notably, the trigger event is now contained twice in the frame.

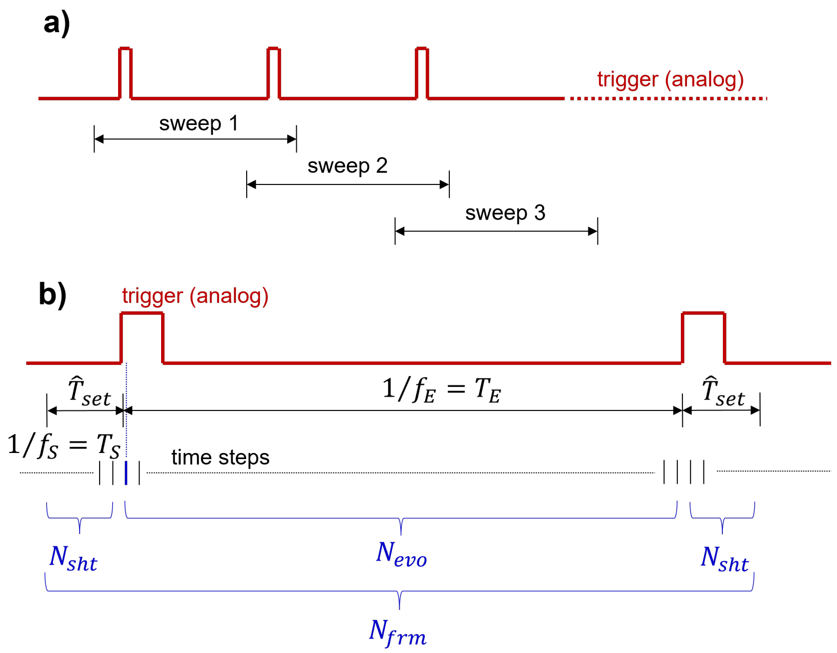

Fig. 1 Overview of the definition of frames for smart averaging. *a)* A train of equally spaced triggers defines a series of sweep frames. These frames contain the evoked window and two shutter intervals (see text). Thus, sweep frames overlap. *b)* A single sweep frame. In the software implementation the frame is aligned to the trigger by choosing the first sample overlapping with the trigger channel (step in blue color). The evoked window (N_{evo} samples) and the two shutters (N_{sht} samples each) are positioned relative to this alignment. Thus, the entire frame contains $N_{frm} = N_{evo} + 2N_{sht}$ samples.

In the current implementation the length of each shutter window was set to 15 ms (N_{sht} =144 samples at 9.6 kHz sampling rate). This value was based on the pulse response of the zero-phase IIR target-band filter described in Section 3. This interval assures that settling effects become negligible.

1.2 Suppression of Electrode Potentials

For suppressing electrochemical electrode potentials a baseline correction is foreseen. It assumes that the evoked signal is near zero ahead of the stimulus. Therefore, the offset of the raw-data in the baseline interval T_Z is computed and subtracted from the entire frame. According to the commutative property of averaging, the baseline correction can be performed after the division by N. The signal-to-noise management (SNM) concept takes advantage of this.

In the current implementation the four samples ahead of the stimulus were chosen for baseline correction. This corresponds to an interval length $T_Z \simeq 0.4\,\mathrm{ms}$.

1.3 Suppression of Myogenic Bursts

We aimed for using N-FTA data [2] for developing a myo-artifact suppression approach (e.g., exclusion of swallowing events). We investigated all 14 data sets and extracted background PSD at 600 Hz (i.e., the center frequency of the HFO target-band). In three of the series (one in subject S4 and two series in subject S7) background PSD was distinctly higher than in the remaining series and visual inspection of raw data confirmed pronounced, isolated muscular artifacts. Therefore, this three series were excluded. The remaining 11 series delivered the following background PSD levels: minimum −25.5 dB, 25 %-percentile −25.1 dB, median −24.9 dB, 75 %-percentile −24.4 dB and maximum −23.7 dB. For providing sum buffer for variation of noise levels in individual sweeps we chose a threshold of −20 dB.

For comparing this threshold to the actual noise level in a single frame the following method was applied. Band-pass filtering was used for assessing the mean power within a spectral band of a frame. Notably, here the output of this filter step is only used for decision about frame acceptance. Since this operation must be carried out at each single sweep, a filter requiring a low computational cost was applied. A four-point moving average decimation filter was chosen as the low-pass component. At 9600 Hz sampling rate a four-point moving average filter delivers an upper corner frequency of 1060 Hz. Four-point decimation reduces sampling rate to 2400 Hz while avoiding aliasing. Thus, it reduces the computational load for the high-pass filter in the next step. This filter was implemented as a second-order Butterworth IIR-filter with 400 Hz lower corner frequency. The evoked interval of the frame was investigated except for the first and last 5 ms (exclusion of the stimulus artifact). Finally, the root-mean-square (rms) voltage of this segment was computed as a surrogate for background power within the segment.

For the obtained 400 to 1060 Hz bandwidth the chosen −20 dB threshold level can be converted to a rms voltage as follows

$$\sqrt{(1060\,\mathrm{Hz} - 400\,\mathrm{Hz})10^{\frac{-20\,\mathrm{dB}}{10}} \times 1\,\mathrm{\mu V}^2\,\mathrm{Hz}^{-1}} = 2.6\,\mathrm{\mu V}. \tag{1}$$

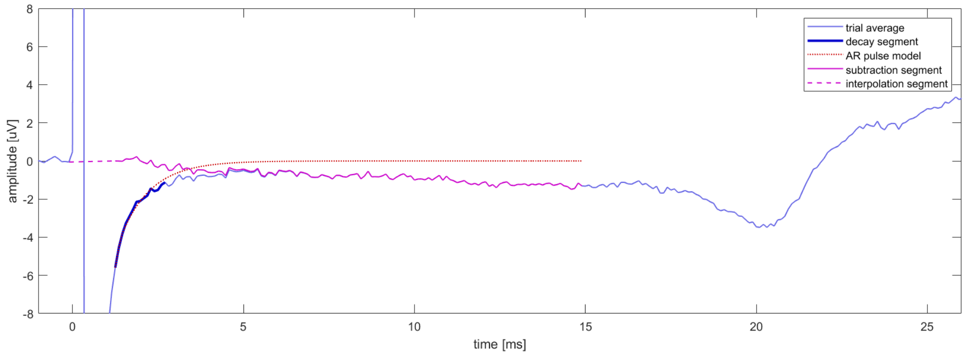

Fig. 2 Example for stimulation spike cancellation in subject S1 ($N = 990, f_E = 3.95\,\text{Hz}$). The input to the cancellation block is the data frame obtained from trial averaging. In a first step the decay segment is identified (dominated by the artifact, bold line). An AR pulse model is fitted to this segment and extrapolated up to 15 ms (dotted line). For cancellation the pulse model is subtracted from the trial average (magenta). In the segment from the last sample ahead of the stimulus to the first sample in the subtraction segment a linear interpolation is performed (dashed line). In this Figure the N20 peak is plotted downwards. See text for details.

Sweeps exceeding this threshold were automatically removed from analysis. This criterion turned out being selective. Only a few percent of sweeps were excluded and sweeps containing visible swallowing artifacts were robustly identified.

2 Stimulation Spike Cancellation

Figure 2 depicts the processing steps of the implemented algorithm. The input is obtained from smart trial averaging. The underlying frame structure is defined in Figure 1.

Decay segment

First a segment dominated by the asymptotic decay of the stimulation artifact is automatically identified. This allows for modeling of the decay by an auto-regressive (AR) linear time independent system. Due to the asymptotic decay of the artifact, a segment early after the stimulus is dominated by the artifact. A flexible choice of interval limits was implemented for choosing the proper segment in individual recordings. The asymptotic segment might have either polarity (positive or negative pulse). In Figure 2 it is negative (i.e., the transient pulse decays upwards towards zero).

A pseudo-code for the choice of the interval limits is listed in Algorithm 1. The first sample in the decay segment was placed 12 samples (i.e., approximately 1.2 ms at 9.6 kHz sampling rate) after the end of the first shutter interval. A variable width of 9 to 48 samples (roughly 1 to 5 ms) was foreseen. The algorithm estimates the signal level p_L at which stochastic noise exceeds the asymptotic decay. The variable p_L is initialized with the first value in the decay segment and pulse polarity is obtained

from this value. Next p_L is updated over the minimum length such that the value closest to zero is stored. Next the decay segment is scanned. When detecting a noise level $< 0.15\,\mu V$ the algorithm stops and returns the last index i_B in the decay interval as the end of the decay segment.

Algorithm 1 Get Decay Segment

procedure DecayEnd(F) ▷ input F ... smart average frame
  int. constants: $N_{sht} = 144; i_A = 12; \check{M} = 9; \hat{M} = 48$ ▷ shutter, 1st index, min. & max. width
  float constants: $\Delta p = 0.15$ ▷ noise tolerance in µV
  —— Initialize Extremum & Artifact Polarity ——
  $p_L \leftarrow F[N_{sht} + i_A]$ ▷ initial low level
  if $p_L < 0$ **then**
    $s \leftarrow -1$ ▷ negative pulse polarity
  else
    $s \leftarrow 1$ ▷ positive pulse polarity
  end if
  —— Update low level over min. width ——
  for $n = 2 : \check{M}$ **do**
    if $s \times F[N_{sht} + i_A + n] < s \times p_L$ **then**
      $p_L \leftarrow F[N_{sht} + i_A + n]$ ▷ update low level
    end if
  end for
  $i_B \leftarrow \check{M}$ ▷ actual width
  —— Update low level over variable width ——
  while $s \times F[N_{sht} + i_B + 1] < s \times p_L + \Delta p$ && $i_B < \hat{M}$ **do**
    $i_B \leftarrow i_B + 1$ ▷ increase width
    if $s \times F[N_{sht} + i_B] < s \times p_L$ **then**
      $p_L \leftarrow F[N_{sht} + i_B]$ ▷ update low level
    end if
  end while
  —— Return last decay segment index ——
  return i_B ▷ return last index
end procedure

AR pulse model

Notably, AR-systems provide an infinite impulse response (IIR) as an approximation of an asymptotic decay. The co-variance algorithm [3] (Matlab function `arcov`, the model order was chosen equal to three) is used for obtaining the auto-regressive polynomial coefficient array $\mathbf{a}$ from the decay segment data. The roots of the polynomial $\mathbf{a}$ were computed [4] (Matlab function `roots`) for fitting the pulse model to the decay segment and performing extrapolation up to 15 ms.

Artifact Cancellation

Within the interval from 1.2 to 15 ms the AR pulse model is subtracted from the trial average. Between the last sample ahead of the stimulus to the first sample in the subtraction segment a linear interpolation is performed. As it was outlined in section 1.1 the smart average frame segment contains two trigger events and, thus, also two trigger artifacts (see main manuscript [1] Figure 2). The identical cancellation is applied to both artifacts.

3 Zero-Phase IIR-Filtering

The target-band band filter applied in this study was originally proposed by Lanzone et al. [5]. While providing excellent filter performance there appears to be a significant inconsistency in terminology and even definition of filter parameters in the literature. This section provides a detailed description of the implementation.

IIR-filter:

Infinite impulse response (IIR) filters are well described in textbooks [3, 6]. Their underlying structure is a discrete linear time independent (LTI) system containing non-zero back-coupling coefficients. The LTI system yields an infinite impulse response but also creates phase-shifts. Notably, IIR-filters are often used for approximating analogue filters (e.g., Butterworth filters) which always produce phase shifts. Such phase shifts alter latency. Thus, zero-phase filters are of advantage.

Zero-Phase filter:

The frequent use of zero-phase IIR filters was pioneered by the MATLAB function `filtfilt`. As the name suggests, this routine performs a double application of an IIR-filter. For avoiding phase shifts, the IIR-filter is applied in a forward time direction in a first step and in a backward time direction in a second step. The forward direction uses the standard IIR transfer function $H_I(\omega)$

$$H_I(\omega) = |H_I(\omega)|e^{j\phi(\omega)}. \tag{2}$$

Here, eq. (2) denotes the transfer function by magnitude and phase ϕ. Application in the backward time direction reverses the sign of phase. Thus, `filtfilt` creates a zero-phase filter H_{I^2}

$$H_{I^2}(\omega) = |H_I(\omega)|e^{j\phi(\omega)}|H_I(\omega)|e^{-j\phi(\omega)} = |H_I(\omega)|^2. \tag{3}$$

Here, phase is canceled. Importunately, the magnitude of the transfer function becomes squared by the double application of the filter. This effect is well described in the MATLAB-documentation. However, no tools are provided which allow for automatic investigation of the effect of squaring on filter parameters. Such investigations can be done manually in MATLAB but this is often overlooked, leading to confusion in literature.

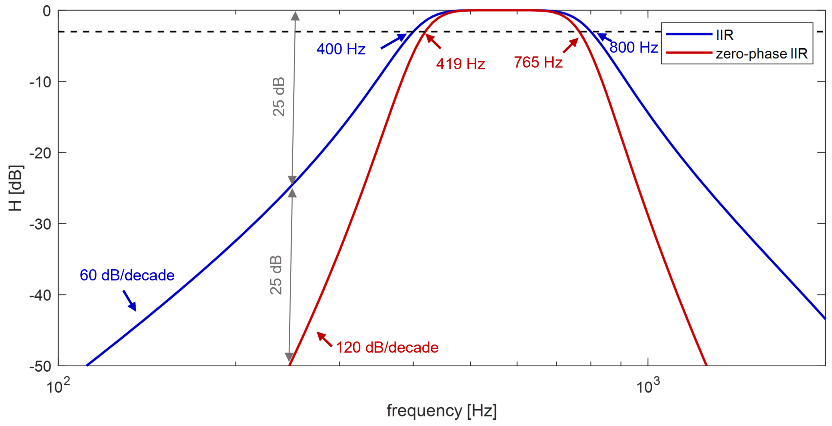

Fig. 3 Magnitude of the transfer functions of the IIR-filter H_I and the corresponding zero-phase IIR-filter H_{I^2} which were applied to the HFO-band. For the zero-phase filter the double application doubles attenuation in dB. For example, near 250 Hz attenuation doubles from 25 dB to 50 dB. In the filter stop-bands double application doubles slope, which corresponds to doubling of filter order. Also filter band-width gets slightly reduced (the dashed line labels the −3 dB corner level).

HFO target-band

Lanzone et al. [5] proposed 400 to 800 Hz Butterworth band-pass for extracting the HFO band. Butterworth filters provide a distinct transition from the pass-band to the stop-band allowing for well defined filtering. Lanzone et al. proposed a filter order of three. Notably, the MATLAB function `butter` (returns the IIR coefficients) applies this filter order to both the lower and the upper corner filter-corner. It is good practice to denote such a band-pass by $2{\times}3^{\text{rd}}$ order[1].

Figure 3 depicts the transfer functions H_I (standard IIR-filter) and H_{I^2} (zero-phase IIR-filter) for the target-band filter in a double logarithmic scale. Importantly, when designing a 400 to 800 Hz 3rd order band-pass, the MATLAB function `filtfilt` applies a 419 to 765 Hz target band filter of $2{\times}6^{\text{th}}$ order. The flat pass band characteristic of Butterworth filters is well maintained. Figure 4 depicts the normalized pulse response of the chosen zero-phase IIR filter.

4 Assessment of Noise Levels and RMS-Amplitude

This section describes the assessment of parameters quantifying noise levels in the raw data and rms-amplitude of evoked HFO responses. Results were obtained from the data of the 14 series.

[1]The statement "2nd order band-pass" does not uniquely distinguish $2{\times}1^{\text{st}}$ order from $2{\times}2^{\text{nd}}$ order.

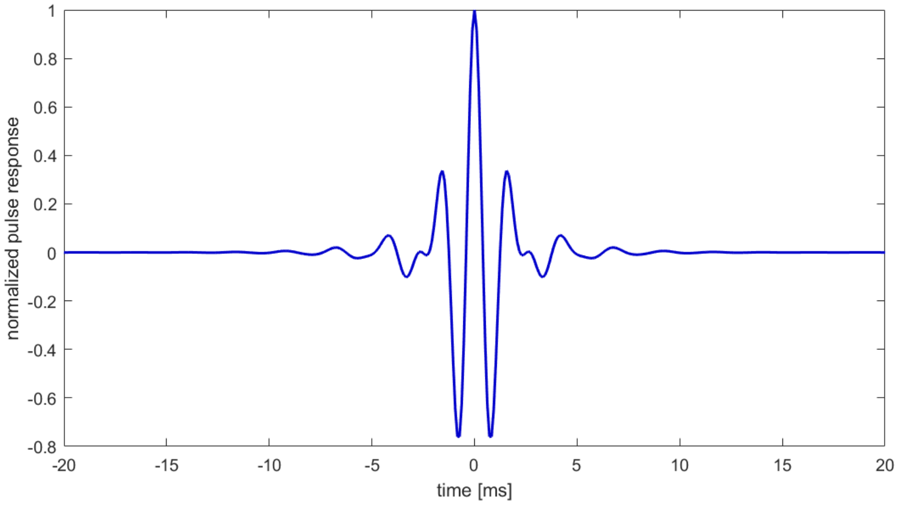

Fig. 4 Normalized pulse response of the target-band filter. Application of the IIR-filter in forward and backward time direction delivers a symmetric pulse response (even function) which is characteristic for zero-phase filters. The pulse settles within less than ±0.15 ms.

Electrode Potentials

For assessing the electrochemical voltages of the electrodes the potential offset in the raw data ahead of each stimulation artifact was assessed. Figure 5 depicts the raw data and the offsets ahead of each stimulus for the series recorded in subject S2 (f_E=3.95 Hz). The mean of these offsets was calculated for quantifying electrode potentials.

Power-Line Interference

For extracting power-line interference stimulation artifacts and linear baseline drift were first removed by preprocessing as described in [2]. Next a zero-phase IIR-band-pass (44.5 to 55.5 Hz, 2×4th order) was applied for obtaining the near 50 Hz interference (Figure 6). For obtaining the peak-value first the rectified mean was computed. This mean was then multiplied by $\pi/2$ for obtaining the mean peak value of the sine wave.

Stimulation Artifact

Smart trial averaging preserves the stimulation artifact while suppressing background activity. Therefore, the output of the smart trial averaging block (see main manuscript [1], Figure 2) was investigated at maximal sweep count (best background suppression). Peak-to-peak artifact amplitude was defined as the difference of the maximal and minimal amplitude within the zero to 5 ms interval. Figure 7 depicts two examples.

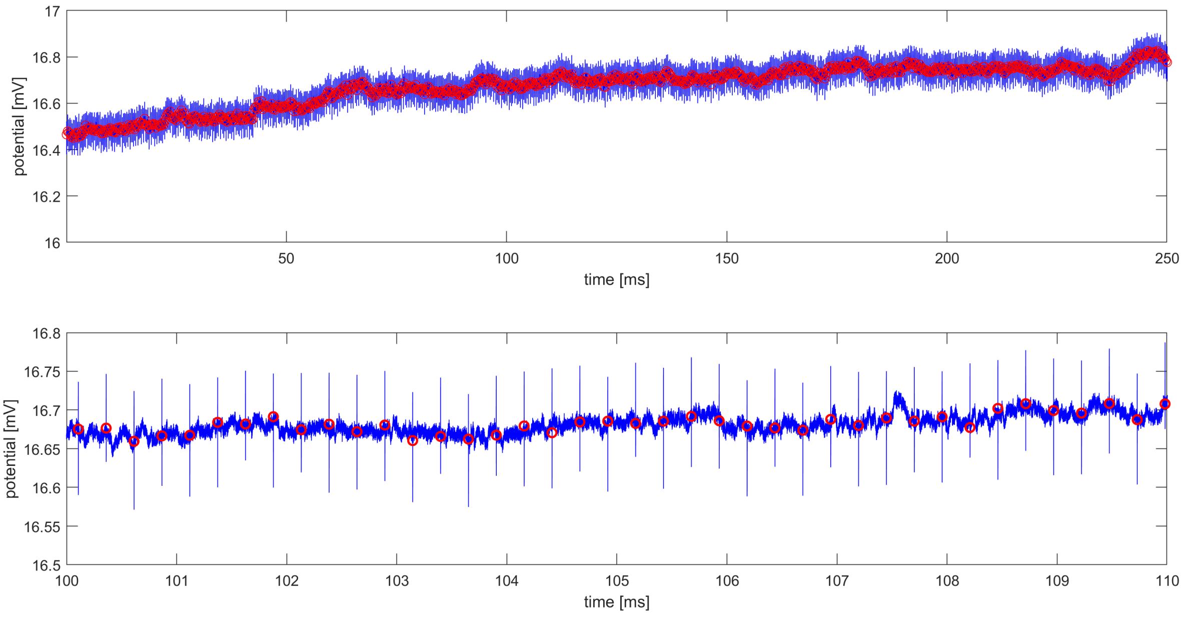

Fig. 5 *Top panel:* Raw data (blue trace) and potential offset ahead of each stimulus (red circles) for subject S2. *Bottom panel:* A 10 s portion of the raw data is magnified for showing stimulation artifacts and markers more clearly. The mean offset in this subject was 16.7 mV. The total drift of the offset over the segment was approximately 0.25 mV.

Equivalent rms-voltage of evoked HFO

As described in [7] and Section 2.3 of the main manuscript [1] SNR-estimation for a trial averaged frame is based on the measurement of the total mean power Σ_W within the response window T_W and the mean background power B_N within the window T_N. The evoked power E_W is then estimated from

$$E_W = \Sigma_W - B_N. \tag{4}$$

The equivalent rms evoked HFO-voltage V_{evo} is then obtained by taking the root

$$V_{evo} = \sqrt{E_W}. \tag{5}$$

5 Testing of Real-Time Signal-to-Noise Management

In [7] a comparison of expected SNR (based on spectral analysis) and SNR observed in real-time trial averaged HFO responses was performed for validation purposes. This section extends this analysis to standard SEPs (N20-P25 component) and investigates computation time of the implementation.

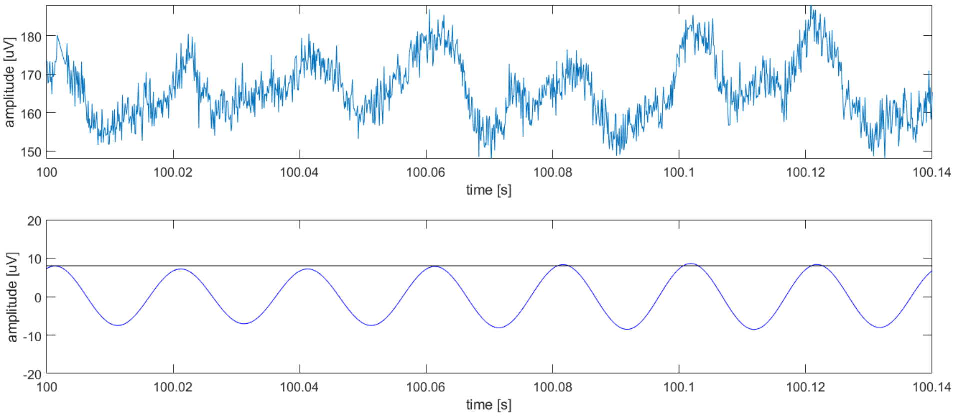

Fig. 6 *Top panel:* A 0.14 s segment of preprocessed raw data obtained from subject S4 (preprocessing removed stimulation artifacts and linear baseline drift as described in [2].) Powerline interference was superimposed on the noisy data. *Bottom panel:* Band-pass filtered power-line interfence (blue trace). The black line depicts the mean peak level (7.24 µV). Thus, the peak-to-peak power-line interference was approximately 14.5 µV. Note that identical scales were applied in both panels.

5.1 Expected and Observed SNR for the N20-P25 Potential

The data of subjects S5 to S10 at a stimulation rate of $f_E = 2.46$ Hz were investigated. As outlined in the main manuscript [1], these recordings allowed for merging of two series. Thus, data sets contained at least 1000 sweeps. The mean evoked-background-ratio (EBR) was assessed for the 20 to 264 Hz target band. We assumed a fixed value of 12 dB for the ratio T_W/T_N in the SNR model. This corresponds to a width of approximately 25 ms of the short latency response. Figure 8 depicts the results.

The largest deviations between model prediction and online SNR were observed for subject S7. As outlined in the main manuscript [1], these data contained significant swallowing artifacts. These artifacts were removed by SNM (evoked response) but were not removed for N-FTA (increased noisy background level). Consequently, response quality exceeded model prediction. After removing this subject from further analysis, a mean correlation coefficient of 0.90 (range 0.65 to 0.99) was observed when comparing the two SNR traces. The rms deviation between the two traces was 1.8 dB (range 0.7 to 2.9 dB). Thus, quantitative analysis confirms good agreement of theoretical predictions and experimental data.

5.2 Implementation and Computation Time

During the development of the SNM concept two versions were tested. Both were implemented in a Matlab environment. Standard Matlab applies a source code interpreter which converts the code on run time into executable processor language. This allows for fast and flexible development at the cost of slowed execution of code.

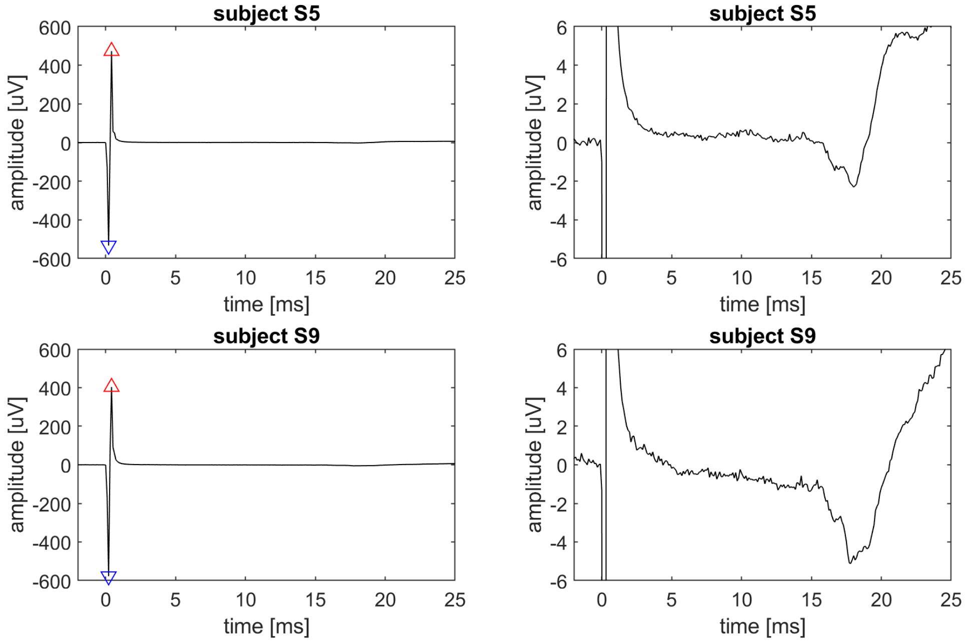

Fig. 7 Stimulation artifacts for two subjects S5 (top row) and S9 (bottom row). In the left column amplitude scale was chosen such, that minimum (blue markers) and maxima (red markers) are depicted. In the right column the scale was reduced by a factor of hundred for making the N20-peak (plotted downwards) visible. Peak-to-peak artifact amplitude was 1010 µV in subject S5 and 980 µV in subject S9.

Therefore, we investigated two options: an implementation using compiled (fast execution) and a version using the interpreter for flexible testing.

Firstly, a prototype allowing for real-time execution during the experiments was developed (analysis of N20-P25 component and HFOs). Here, the g.USBamp (g.tec GmbH, Austria) biopotential amplifier provides an interface to the Matlab plug-in Simulink. A C-MEX S-function wrapper was used for linking a GNU-complied SNM-function to Simulink. Briefly, this SNM-wrapper read the raw data from the g.USBamp interface, performed all signal processing operations, and passed the results to standard Matlab visualization (SEP-traces labeled with actual SNR values). This implementation did not allow for assessing the run-time percentage for the wrapper application on the processor. However, when applying this prototype for data acquisition in subjects S5 to S10, run-time was always sufficiently small allowing for online display of evoked responses and signal-quality without any noticeable delay.

Since the development of the above mentioned Simulink block was based on a subset of data (subjects S1 to S3) also a real-time capable off-line standard Matlab

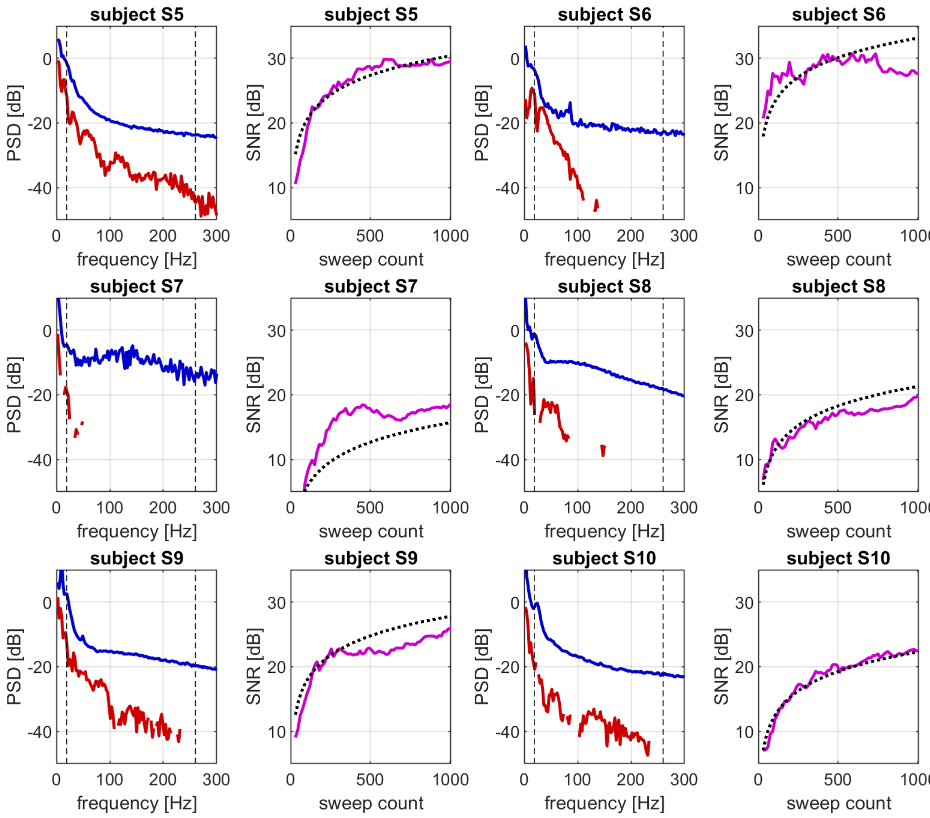

Fig. 8 *Columns 1 and 3:* N-FTA performed up to the high-γband for subjects S5 to S10. The blue traces depict background activity. The red traces reflect stimulus locked evoked activity. PSD on the y-axis was converted to dB. *Columns 2 and 4:* SNR obtained from real-time capable SNM (magenta) and N-FTA (dotted line). See also text.

implementation was developed which allowed for further optimization of software parameters based on all collected data (subjects S1 to S10). This version was also used for analysis of the results presented in the paper. Here, computation time for analyzing 990 sweeps was less than 3 s on a standard laptop. Notably, recording of this sweep count at the high stimulation rate (3.95 Hz) takes 250 s. Thus, even when using the relatively slow Matlab interpreter, evaluation of signal processing for a single channel requires only about 1 % of the acquisition time.

References

[1] Fischer, G., Holzknecht, M.E., Hauheisen, J., Baumgarten, D., Kofler, M.: A Real-Time Signal-To-Noise Management Approach for Evoked Potentials – Assessing and Explaining Response Quality. submitted to Bioengineering (2026)

[2] Fischer, G., Haueisen, J., Baumgarten, D., Kofler, M.: Spectral separation of evoked and spontaneous cortical activity, part 1: Delta to high gamma band. Biomedical Signal Processing and Control **92**, 106094 (2024) https://doi.org/10.1016/j.bspc.2024.106094

[3] Stoica, P., Moses, R.: Spectral Analysis of Signals. Prentice-Hall International, Inc., New Jersey (2005)

[4] Press, W.H., Teukolsky, S.A., Vetterling, W.T., Flannery, B.P.: Numerical Recipes - The Art of Scientific Computing. Cambridge University Press, 3rd ed. New York (2007)

[5] Lanzone, J., Boscarino, M., Ricci, L., Insola, A., Tombini, M., Di Lazzaro, V., Assenza, G.: The effects of antiepileptic drugs on high-frequency oscillations in somatosensory evoked potentials. Clinical Neurophysiology **131**(8), 1917–1924 (2020) https://doi.org/10.1016/j.clinph.2020.05.022

[6] Proakis, J.G., Manolakis, D.G.: Digital Signal Processing - Priciples, Algorithms and Applications. Prentice-Hall International, Inc., 3rd ed. New Jersey (2013)

[7] Fischer, G., Haueisen, J., Baumgarten, D., Kofler, M.: Converting spectral evoked-to-background ratio into time-domain signal-to-noise ratio – validation for high frequency oscillations. MethodsX, 103617 (2025) https://doi.org/10.1016/j.mex.2025.103617
